# Expression of miRNA-122 Induced by Liver Toxicants in Zebrafish

**DOI:** 10.1155/2016/1473578

**Published:** 2016-08-03

**Authors:** Hyun-Sik Nam, Kyu-Seok Hwang, Yun-Mi Jeong, Jeong-Im Ryu, Tae-Young Choi, Myung-Ae Bae, Woo-Chan Son, Kwan-Hee You, Hwa-Young Son, Cheol-Hee Kim

**Affiliations:** ^1^New Drug Development Center, Osong Medical Innovation Foundation, Chungbuk 28160, Republic of Korea; ^2^Graduate School of New Drug Discovery and Development, Chungnam National University, Daejeon 34134, Republic of Korea; ^3^Department of Biology, Chungnam National University, Daejeon 34134, Republic of Korea; ^4^Department of Drug Discovery Platform Technology, Korea Research Institute of Chemical Technology (KRICT), Daejeon 34114, Republic of Korea; ^5^Department of Pathology, Asan Medical Center, University of Ulsan College of Medicine, Seoul 05505, Republic of Korea; ^6^College of Veterinary Medicine, Chungnam National University, Daejeon 34134, Republic of Korea

## Abstract

MicroRNA-122 (miRNA-122), also known as liver-specific miRNA, has recently been shown to be a potent biomarker in response to liver injury in mammals. The objective of this study was to examine its expression in response to toxicant treatment and acute liver damage, using the zebrafish system as an alternative model organism. For the hepatotoxicity assay, larval zebrafish were arrayed in 24-well plates. Adult zebrafish were also tested and arrayed in 200 mL cages. Animals were exposed to liver toxicants (tamoxifen or acetaminophen) at various doses, and miRNA-122 expression levels were analyzed using qRT-PCR in dissected liver, brain, heart, and intestine, separately. Our results showed no significant changes in miRNA-122 expression level in tamoxifen-treated larvae; however, miRNA-122 expression was highly induced in tamoxifen-treated adults in a tissue-specific manner. In addition, we observed a histological change in adult liver (0.5 *μ*M) and cell death in larval liver (5 *μ*M) at different doses of tamoxifen. These results indicated that miRNA-122 may be utilized as a liver-specific biomarker for acute liver toxicity in zebrafish.

## 1. Introduction

During the process of drug development, many compounds fail somewhere along the pipeline due to drug-induced toxicity. Half of these compounds are removed due to hepatotoxicity, including necrosis, steatosis, cholestasis, proliferation, inflammation, and bile duct hyperplasia [1]. Drug-induced liver injury (DILI) has been established as a major cause of acute liver failure in the United States. DILI further represents a major reason why approved drugs are restricted or removed from the market [2, 3]. Thus, rapid and accurate detection of hepatotoxicity remains a vital issue in new drug development. Over the years, various model systems have been developed to enable detection of potential liver toxicity of chemicals and drugs [4]. The strategy of screening large groups of chemical compounds in cell culture for their effects on specific cellular characteristics has already been well established; however, many biological processes cannot be reproduced in cultured cells and often require the three-dimensional environment of cells to determine their function. Furthermore, metabolism of compounds in whole organisms may be profoundly different from the processes at work in vitro. It is desirable therefore to screen large numbers of small molecule compounds for biological activity in whole organisms with high throughput as early as possible in the screening process [5, 6]. Due to transparent embryos and rapid organogenesis, zebrafish have been recognized as a tool for developmental biologists and researchers in a wide variety of fields. In comparison to rodents and nonhuman primates, there are numerous advantages to the use of zebrafish as a toxicological model species [7, 8], and the number of publications utilizing zebrafish has increased dramatically in recent years. The main benefits of zebrafish as a toxicological model over other vertebrate species are well established regarding size, morphology, and easy and cost-effective husbandry and maintenance conditions [9].

MicroRNAs (miRNAs) are small noncoding RNAs that posttranscriptionally regulate gene expression [10]. It has been estimated that over 60% of human protein-coding genes are regulated by miRNAs [11]. In addition, miRNAs released from cells are capable of regulating gene translation in distant cells in a process similar to cell-to-cell communication by cytokines and other soluble factors [12]; however, the roles of miRNAs are not yet fully understood [13]. As a liver-specific miRNA species, miRNA-122 has recently shown potential as a predictor of liver injury and thus could serve as an addition to the repertoire of standard hepatic injury biomarkers used in humans [14] and rodents [15]. Studies have been performed to assess whether the cellular release of miRNA-122 is associated with specific types of liver injury [16–18] and, due to their numerous functions and cellular specificity, miRNAs are potential biomarkers for particular conditions and cell types.

In the present study, we investigated the expression of miRNA-122 during the initiation and progression of acute liver injury induced by liver toxicants. In addition, we assessed the usefulness of the zebrafish assay model in early-stage preclinical liver toxicity screenings.

## 2. Materials and Methods

### 2.1. Maintenance of Zebrafish

Zebrafish (*Danio rerio*) were maintained and raised under standard conditions as previously described [9]. All experiments were approved by the Institutional Animal Care and Use Committees (IACUC) of Chungnam National University (CNU-00620).

### 2.2. Liver Toxicants Treatment

Tamoxifen (T5648, Sigma-Aldrich) or acetaminophen (A7085, Sigma-Aldrich [19]) was dissolved in DMSO. For the hepatotoxicity assay, 4 dpf (day after fertilization) zebrafish larvae were arrayed in 24-well plates (five individuals per well) containing 1 mL embryonic medium that was diluted from 1000x stock solutions of NaCl (29.4 g/100 mL, Sigma-Aldrich), KCl (1.27 g/100 mL, Sigma-Aldrich), CaCl_2_·2H_2_O (4.85 g/100 mL, Sigma-Aldrich), and MgSO_4_·7H_2_O (8.13 g/100 mL, Sigma-Aldrich), as these solutions can be autoclaved and stored at room temperature. The larvae were exposed to various doses of liver toxicants for 24 hours. For imaging, larvae were anesthetized with tricaine (MS-220, Sigma-Aldrich) and mounted on 3% methyl cellulose (Sigma-Aldrich). Mounted larvae were imaged with a Leica MZ APO stereomicroscope and DC300 FX (Leica, Japan). In addition, 3-month-old zebrafish were arrayed in 200 mL cages (3 individuals per cage) and exposed to liver toxicants for 24 hours. To induce nervous system-specific toxicity, we used the nitroreductase/metronidazole system in combination with the neuron-specific transgenic line. Nitroreductase converts the nontoxic prodrug, metronidazole (Mtz), into cytotoxic agents [20].

### 2.3. Blood Concentration Analysis of Liver Toxicant

Blood (7–10 *μ*L/fish) was collected from adult zebrafish by tail cutting. Each zebrafish blood sample (3 *μ*L) was placed in a 1.5 mL microfuge tube and mixed with 72 *μ*L of solution (5 ng/mL disopyramide in ACN) to precipitate plasma proteins and protein from cells such as red blood cells. The mixture was vigorously mixed for 10 min using a vortex mixer, followed by centrifugation at 10,000 ×g for 10 min at 4°C using a temperature-controlled microcentrifuge (Eppendorf, Hamburg, Germany). After the mixture was transferred to a fresh vial, an aliquot of 50 *μ*L of each supernatant was directly injected into the LC-MS/MS system for analysis.

A 1200-series HPLC system coupled to an Agilent 6460 triple-quadrupole mass spectrometer (Agilent, CA, USA) was operated under positive-ionization mode for the LC-MS/MS analysis. Spectrometry was performed using multiple-reaction monitoring mode. The LC chromatograph was equipped with a Hypersil Gold C18 column (100 × 2.1 mm; i.d., 3 *μ*m; Thermo, Waltham, MA, USA) and maintained at 35°C. The mobile phase for sample analysis consisted of acetonitrile/10 mM ammonium formate in water (80 : 20, v/v) at a flow rate of 0.3 mL/min.

### 2.4. Quantitative Real-Time RT-PCR of miRNA-122

miRNA-122 stem-loop sequences of vertebrates obtained from microRNA database (http://www.mirbase.org/) were aligned and analyzed using ClustalX (version 2.0) and GeneDoc (version 2.7.000) software. Small RNAs were isolated from the livers of drug-treated adult zebrafish using the mirVana^TM^ miRNA Isolation kit (AM1560, Ambion). Subsequently, we synthesized cDNA from purified small RNAs using the TaqMan MicroRNA Reverse Transcription Kit (4366596, Applied Biosystems). Specific primers targets were hsa-miRNA-122 (stem-loop accession MI0000442) and U6 snRNA (NCBI accession #NR_004394). U6 snRNA was used as an endogenous control for normalization. For quantitative RT-PCR, we used TaqMan Universal PCR Master Mix II and No UNG (4440043, Applied Biosystems) and performed quadruplication per cDNA. The relative expression of the target gene was calculated using a 7500 Fast Real-Time PCR System and its associated software (version 2.0.1, Applied Biosystems) [15].

miRNA-122 expression was analyzed in the liver, brain, heart, and intestine, to verify the sensitivity and specificity of miRNA-122 as a liver injury biomarker.

### 2.5. Histopathology

After tamoxifen treatment, 3-month-old zebrafish were fixed using 10% neutral-buffered formalin and sectioned longitudinally for histopathologic evaluation. The livers were embedded in paraffin and sectioned at 5 *μ*m thickness and stained with hematoxylin and eosin.

### 2.6. Statistical Analysis

Data of miRNA-122 expression was indicated as mean ± standard error (SE). Data of DMSO- and toxicants-treated groups were analyzed in a one-way analysis of variance (ANOVA). The significance of differences between DMSO- and tamoxifen-treated groups was assessed by Dunnett's multiple comparison tests using GraphPad Prism for Windows (version 6.0). Significance between DMSO- and toxicants-treated groups is indicated as ^*∗*^ *p* < 0.05 or ^*∗∗*^ *p* < 0.01.

## 3. Results

### 3.1. Toxicity in Zebrafish Larvae and Adult Induced by Liver Toxicants

**Figure 1:**
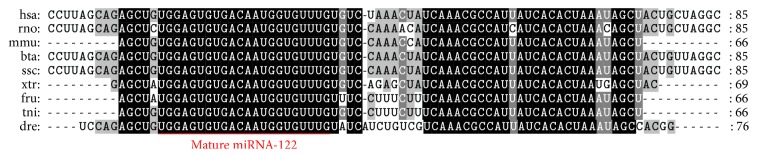
Alignment of miRNA-122 stem-loop sequences among vertebrates. hsa:* Homo sapiens* (MI0000442); rno:* Rattus norvegicus* (MI0000891); mmu:* Mus musculus* (MI0000256); bta:* Bos taurus* (MI0005063); ssc:* Sus scrofa* (MI0002413); xtr:* Xenopus tropicalis* (MI0004824); fru:* Fugu rubripes* (MI0003315); tni:* Tetraodon nigroviridis* (MI0003316); dre:* Danio rerio* (MI0001965).

**Figure 2:**
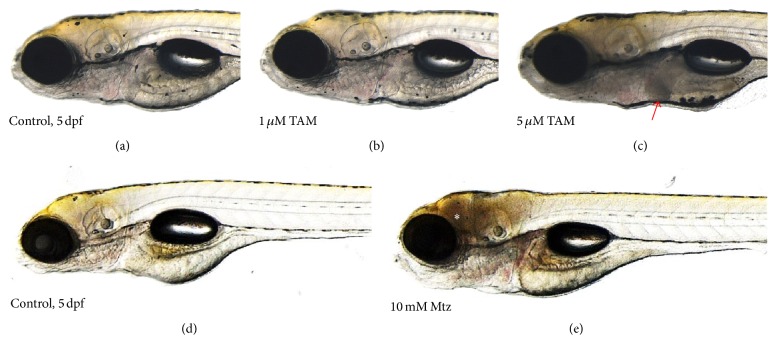
Tissue-specific cell death in the zebrafish larvae treated with tamoxifen (TAM) or metronidazole (Mtz). (a) 0.1% DMSO-treated control (5 dpf) and (b) 1 *μ*M and (c) 5 *μ*M TAM-treated zebrafish larvae. (d) 0.1% DMSO-treated control (2 dpf) and (e) 10 mM Mtz-treated zebrafish larvae. Liver-specific cell death was visualized by reduction of transparency in the TAM-treated zebrafish larvae (red arrow), compared to brain-specific cell death in the Mtz-treated larvae (white asterisk). For Mtz experiments, the transgenic zebrafish system, having neuron-specific nitroreductase expression, was used [20].

**Figure 3:**
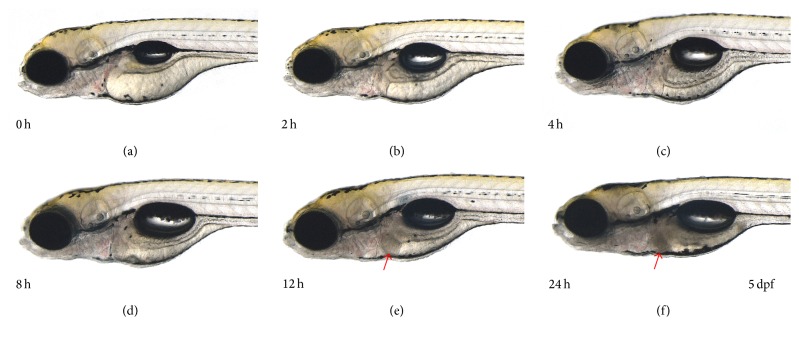
Duration-dependent changes induced by exposure to 5 *μ*M tamoxifen in zebrafish larvae. (a) Pretreatment at 4 dpf and (b) 2-hour exposure, (c) 4-hour exposure, (d) 8-hour exposure, (e) 12-hour exposure, and (f) 24-hour exposure. After 12 hours, tamoxifen induced cell death in zebrafish larvae liver.

**Figure 4:**
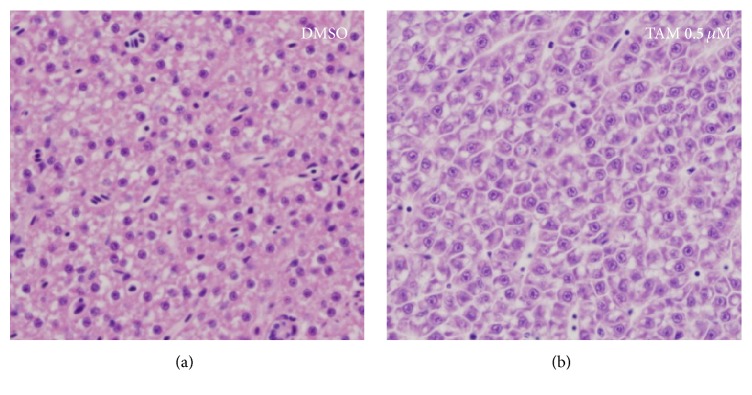
Histopathology of the adult liver treated with 0.5 *μ*M tamoxifen for 24 hours. (a) Control untreated zebrafish liver and (b) 0.5 *μ*M tamoxifen-treated zebrafish liver. Any significant cell death was not detectable in the 0.5 *μ*M tamoxifen-treated adult liver, but vacuole formation was detected in hepatocytes. H&E staining.

The liver-specific miRNA-122 sequence was shown to be highly conserved between various species (Figure 1). Zebrafish larvae (4 dpf) were utilized to test the toxicity in the liver. In our previous report [20], severe cell death in zebrafish was visualized as a reduction of transparency following metronidazole treatment. This method allowed simple detection of the damaged tissue or organ in toxicant-treated larvae. When larvae were treated with 5 *μ*M tamoxifen for 24 hours (h), a reduction of liver transparency was observed which indicated liver toxicity (Figure 2). To explore the effects of treatment duration in tamoxifen-induced liver toxicity, zebrafish larvae were treated with 5 *μ*M tamoxifen for up to 24 h. Tamoxifen-induced liver toxicity in the larvae was visually detected as early as 12 h (Figure 3). In addition to larvae, 3-month-old adult zebrafish were treated with 0.5 *μ*M tamoxifen for 24 h. Although tamoxifen-treated liver exhibited vacuoles in hepatocytes, we could not detect dead and/or dying cells (Figure 4) suggesting that tamoxifen induced liver damage but did not cause cell death in 0.5 *μ*M tamoxifen-treated adult zebrafish.

### 3.2. Quantitative Expression Levels of miRNA-122

**Figure 5:**
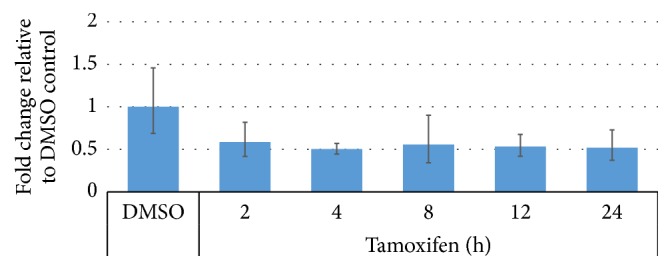
Quantitative expression levels of miRNA-122 in zebrafish larvae (5 dpf). Expression of miRNA-122 did not change as a result of 5 *μ*M tamoxifen in zebrafish larvae. Error bars indicate minimum and maximum values of relative quantification.

**Figure 6:**
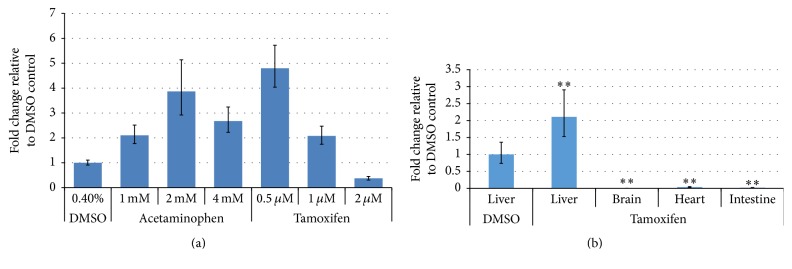
Quantitative expression levels of miRNA-122 in liver toxicant-treated adult zebrafish. (a) Quantitative expression levels of miRNA-122 in tamoxifen- (or acetaminophen-) treated adult zebrafish livers. miRNA-122 expression was upregulated at 0.5 *μ*M and 1 *μ*M and downregulated at 2 *μ*M tamoxifen. miRNA-122 expression was gradually upregulated at 1 mM and 2 mM and decreased at 4 mM acetaminophen. (b) Tissue-specific expression of miRNA-122. miRNA-122 was expressed in the liver of tamoxifen-treated zebrafish, but not in the brain, heart, and intestine at 0.5 *μ*M. Error bars indicate minimum and maximum values of relative quantification (^*∗∗*^ *p* < 0.01).

To measure the effect of liver toxicants on miRNA-122 expression, we carried out quantitative RT-PCR from whole larvae samples treated with 5 *μ*M tamoxifen for 2- to 24-hour time duration. Tamoxifen-treated zebrafish larvae showed no increase in miRNA-122 expression levels (Figure 5). miRNA-122 expression levels were also examined in adult zebrafish. Adult zebrafish were treated with various doses of liver toxicants (tamoxifen or acetaminophen) for 24 hours and analyzed for miRNA-122 expression levels using quantitative RT-PCR on dissected livers. The data showed miRNA-122 expression levels gradually increasing in acetaminophen- and tamoxifen-treated animals at low doses and gradually decreasing at high doses (Figure 6(a)). To confirm the tissue-specific pattern of miRNA-122 expression, quantitative RT-PCR was performed on various dissected tissues including liver, brain, heart, and intestine of 0.5 *μ*M tamoxifen-treated adult zebrafish. We found that miRNA-122 exhibited a liver-specific expression pattern while being only marginally detected in the brain, heart, and intestine of tamoxifen-treated zebrafish (Figure 6(b)).

### 3.3. Blood Concentration of Liver Toxicant

Bioanalysis using LC-MS/MS was performed to correlate the induced phenotype with the actual concentration of the tested compound. The tamoxifen-treated group showed a dose-dependent increase of tamoxifen concentration in blood (Table 1).

## 4. Discussion

New drug discovery and development are a protracted and uncertain process. Many drugs fail during development and are rejected due to unacceptable toxicity. Thereby, predictive assays for toxicity are an important part of the drug development process. Due to the practical and ethical concerns associated with human experimentation, animal models have been essential in evaluating drug safety. Indeed, animal and in vitro models serve as an important resource for toxicity and safety information, but emerging alternative translational approaches may eventually provide an important link between in vitro studies and mammalian animals models [21]. In vivo zebrafish assays have proven to be a rapid, cost-efficient, and reliable toxicity model to serve as the intermediate step between cell-based and mammalian testing [22]. In addition, the high homology of miRNA-122 among human, rodent, and zebrafish (Figure 1) supports the utilization of the zebrafish model as a suitable alternative to mammalian species in liver toxicity screenings.

The liver is involved in a number of vital activities including metabolism, detoxification, and maintenance of homeostasis. Drug-induced liver injury is a leading cause of drug attrition during drug development and postmarketing drug withdrawal [23–25]. Hepatotoxicity is a potential adverse side effect of the drug tamoxifen [26] which is an estrogen-receptor antagonist used in the treatment and prevention of hormone-dependent breast cancer. Tamoxifen causes hepatic steatosis in a significant number of patients that can progress toward steatohepatitis [27]. In this study, we analyzed the concentration of tamoxifen in zebrafish and found that the blood concentration of tamoxifen increased in a dose-dependent manner in the tamoxifen-treated group. Tamoxifen may continually accumulate in the blood for up to 24 hours after treatment. Initially, we considered whether or not chemically amended water was a suitable method of treatment exposure for zebrafish but the results demonstrated that tamoxifen could indeed enter the zebrafish via water (Table 1).

Macroscopically, larvae showed tissue-specific cell death (pale discoloration) in the liver at 5 *μ*M tamoxifen (Figure 2). At the same concentration, the liver of tamoxifen-treated larvae showed duration-dependent pale discoloration (Figure 3), starting at 12 h. However, we could not find evidence of cell death in adults (Figure 4). These results were inconsistent with the results of the miRNA-122 assay and blood concentration measurements and may suggest that zebrafish absorbed tamoxifen from the water but that low doses and short-term application were not sufficient to induce morphological changes in the target organ of adult zebrafish. In adults, miRNA-122 expression was significantly increased at 0.5 *μ*M (Figure 6(a)) and this increase was isolated to the liver (Figure 6(b)). Thus, miRNA-122 expression changes were detected for short-duration and low dose of toxicants prior to the onset of morphological changes in the injury process. High dose (2 *μ*M) significantly downregulated miRNA-122 expression, while low and medium doses (0.5 *μ*M and 1 *μ*M) upregulated expression. It is possible that induction of miRNA-122 expression levels in 0.5 *μ*M tamoxifen-treated adults is caused by hepatocytes damage in the liver and not by cell death, suggesting that an increase in miRNA-122 expression in the liver may accompany hepatocyte damage. This warrants further mechanism studies which are currently underway in our laboratory.

Recently, many studies have shown that miRNAs are differentially expressed in various tissues during normal physiology, injuries, or diseases. The cellular level of a given miRNA may be increased or decreased following drug exposure, leading to corresponding changes in the amount of miRNA released from the cells. miRNA release processes involving microparticles, exosomes, and protein complexes may be altered during exposure. miRNA expression patterns identified to date have not been subject to rigorous qualification and thus remain unproven [28–30]; however, it has been found that miRNA-122 expression levels in the liver [31] and circulating levels of miRNA-122 are significantly increased in mice after treatment with acetaminophen [31],* d*-galactosamine, or ethanol [14].

In this study, the change of miRNA-122 expression was easily and rapidly detectable. Assessment of zebrafish larvae showed that the tamoxifen-treated group did not exhibit increased miRNA-122 expression (Figure 5). In developing zebrafish embryos, miRNA-122 is required for normal hepatic cell differentiation [32]; however, miRNA-122 expression was noted as being consistently high in the liver during exposure to tamoxifen without exhibiting dose-dependence. This finding also correlated with the blood concentration data of tamoxifen. The increase of miRNA-122 expression in adult zebrafish treated with tamoxifen was likely due to hepatocellular damage in the liver. Also, the tamoxifen-treated zebrafish demonstrated increased expression in the liver, but not in the brain, heart, and intestine (Figure 6(b)), indicating the potential of miRNA-122 as a sensitive liver-specific biomarker to assess liver toxicity. Increased miRNA-122 expression at low dose of toxicants also suggests that miRNA-122 may serve as an early biomarker of liver injury prior to the onset of morphological changes.

Interestingly, we found a very high level of tamoxifen concentration in the blood (Table 1) which was 16~20-fold higher than concentration administrated in the water environment of fish tank. Currently, we have no clear explanation of this unexpected high accumulation of chemical compound in the blood of treated adult zebrafish; however, we speculate that this actual concentration in the body may explain the different effective concentrations in liver toxicity between early zebrafish larvae (5 *μ*M tamoxifen, Figure 2(c)) and adult zebrafish (0.5 *μ*M tamoxifen, Figure 6(a)). In addition, this result may also explain why induced miRNA-122 expression begins to decline from the relatively low concentration of 0.5 *μ*M tamoxifen (8 *μ*M in the body) to higher doses of 1 *μ*M and 2 *μ*M (20 *μ*M and 34 *μ*M in the body, resp.) (Figure 6(a)). However, to verify these speculations we will need to conduct further experiments in following mechanism studies.

## 5. Conclusion

The objective of this study was to measure miRNA-122 expression in zebrafish during acute liver toxicity in order to determine the value of miRNA-122 expression as a biomarker for liver injury in this species. After the exposure of toxicants in the zebrafish, miRNA-122 was specifically expressed in the liver. The findings support miRNA-122's potential as a biomarker for acute liver toxicity applicable to the assessment of liver toxicity during drug development. Also, the zebrafish system demonstrated viability as an alternative animal model in the assessment of drug toxicity.

## Figures and Tables

**Table 1:** Blood concentration of tamoxifen-treated zebrafish.

Group	Blood concentration
	Average ± SE (fold change between water and blood concentration)
Control	0.00 ± 0.0 *μ*M
Low group (0.5 *μ*M)	8.01 ± 0.5 *μ*M (~16-fold)
Middle group (1 *μ*M)	20.4 ± 1.3 *μ*M (~20-fold)
High group (2 *μ*M)	34.1 ± 4.6 *μ*M (~17-fold)
