# Corrigendum to “Expression of miRNA-122 Induced by Liver Toxicants in Zebrafish”

**DOI:** 10.1155/2017/1347806

**Published:** 2017-08-02

**Authors:** Hyun-Sik Nam, Kyu-Seok Hwang, Yun-Mi Jeong, Jeong-Im Ryu, Tae-Young Choi, Myung-Ae Bae, Woo-Chan Son, Kwan-Hee You, Hwa-Young Son, Cheol-Hee Kim

**Affiliations:** ^1^New Drug Development Center, Osong Medical Innovation Foundation, Chungbuk 28160, Republic of Korea; ^2^Graduate School of New Drug Discovery and Development, Chungnam National University, Daejeon 34134, Republic of Korea; ^3^Department of Biology, Chungnam National University, Daejeon 34134, Republic of Korea; ^4^Department of Drug Discovery Platform Technology, Korea Research Institute of Chemical Technology (KRICT), Daejeon 34114, Republic of Korea; ^5^Department of Pathology, Asan Medical Center, University of Ulsan College of Medicine, Seoul 05505, Republic of Korea; ^6^College of Veterinary Medicine, Chungnam National University, Daejeon 34134, Republic of Korea

In the article titled “Expression of miRNA-122 Induced by Liver Toxicants in Zebrafish” [1], there was an error in Materials and Methods, where the subtitle “2.4. Quantitative Real-Time RT-PCR of miRNA-122,” should be corrected to “2.4. Quantitative Real-Time PCR of miRNA-122.” In addition, there is an error in the legend of Figure 2(d), which should be corrected as follows.

## Figures and Tables

**Figure 2:**
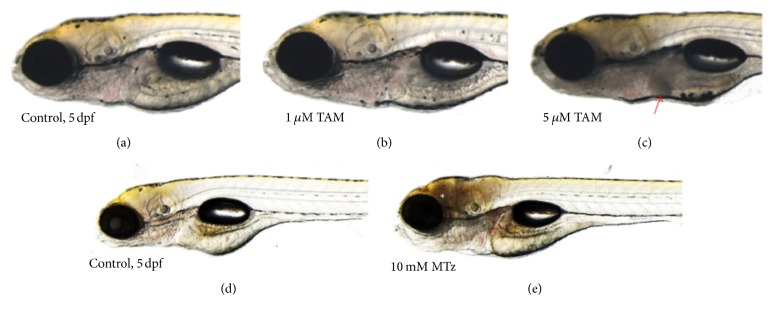
Tissue-specific cell death in the zebrafish larvae treated with tamoxifen (TAM) or metronidazole (Mtz). (a) 0.1% DMSO-treated control (5 dpf) and (b) 1 *μ*M and (c) 5 *μ*M TAM-treated zebrafish larvae. (d) 0.1% DMSO-treated control (5 dpf) and (e) 10 mM Mtz-treated zebrafish larvae. Liver-specific cell death was visualized by reduction of transparency in the TAM-treated zebrafish larvae (red arrow), compared to brain-specific cell death in the Mtz-treated larvae (white asterisk). For Mtz experiments, the transgenic zebrafish system, having neuron-specific nitroreductase expression, was used [20].
